# Risk factors analysis of surgical complications of hepatic hemangioma: a modified Clavien-Dindo classification-based study

**DOI:** 10.1186/s12893-023-02009-3

**Published:** 2023-05-06

**Authors:** Kai Yang, Yan Ma, Zelong Yang, Yanling Yang, Wenjie Song, Weigang Chen, Weihao Lv, Ruohan Zhang, Yong Chen, Hongyu Qiao

**Affiliations:** 1 Department of Hepatobiliary Surgery, Xi Jing Hospital, Air Force Medical University, Xi’an, 710032 China; 2 Department of Gynecology and Obstetrics, Xi Jing Hospital, Air Force Medical University, Xi’an, 710032 China; 3 Department of Neurosurgery, Xi Jing Hospital, Air Force Medical University, Xi’an, 710032 China; 4 Department of Pediatrics, Xi Jing Hospital, Air Force Medical University, Xi’an, 710032 China

**Keywords:** Hepatic hemangioma, Surgery, Clavien-Dindo classification, Risk factor, Complication

## Abstract

**Purpose:**

There are few studies on the risk factors of postoperative complications after surgical treatment of hepatic hemangioma (HH). This study aims to provide a more scientific reference for clinical treatment.

**Methods:**

The clinical characteristics and operation data of HH patients undergoing surgical treatment in the First Affiliated Hospital of Air Force Medical University from January 2011 to December 2020 were retrospectively collected. All enrolled patients were divided into two groups based on the modified Clavien-Dindo classification: Major group (Grade II/III/IV/V) and Minor group (Grade I and no complications). Univariate and multivariate regression analysis was used to explore the risk factors for massive intraoperative blood loss (IBL) and postoperative Grade II and above complications.

**Results:**

A total of 596 patients were enrolled, with a median age of 46.0 years (range, 22–75 years). Patients with Grade II/III/IV/V complications were included in the Major group (*n* = 119, 20%), and patients with Grade I and no complications were included in the Minor group (*n* = 477, 80%). The results of multivariate analysis of Grade II/III/IV/V complications showed that operative duration, IBL, and tumor size increased the risk of Grade II/III/IV/V complications. Conversely, serum creatinine (sCRE) decreased the risk. The results of multivariate analysis of IBL showed that tumor size, surgical method, and operative duration increased the risk of IBL.

**Conclusions:**

Operative duration, IBL, tumor size, and surgical method are independent risk factors that should be paid attention to in HH surgery. In addition, as an independent protective factor for HH surgery, sCRE should attract more attention from scholars.

## Introduction

Hepatic hemangioma (HH) in adults is the most common benign liver tumor, with an incidence of up to 7% [1–3]. With the popularization of physical examination and the development of imaging examination, the detection rate of HH has increased year by year. Most HH is less than 3 cm when detected, and only a minority of patients have clinical symptoms. The presence or absence of symptoms depends on the size and location of the HH [3, 4]. Generally, HH is considered to be vascular malformation caused by excessive vascular development or abnormal differentiation during embryonic development [5]. And it may be related to endogenous and exogenous female sex hormones [6], which may be one of the reasons for the high incidence of this disease in women. The tumor is mainly composed of a large amount of vascular tissue, separated from the normal liver parenchyma by a fibrous sheath, usually supplied by the hepatic artery [5, 7]. Actually, HH is a benign liver disease with no obvious malignant tendency and can coexist with patients for life. However, some patients still have symptoms such as abdominal pain or spontaneous rupture due to the progression or compression of the HH [7]. Therefore, this potentially fatal risk is why these symptomatic patients need treatment.

The diagnosis of HH mainly relies on imaging examinations, and the combined application of these imaging examinations can greatly improve diagnostic accuracy. Ultrasound is routinely preferred [8], in addition to computed tomography (CT), magnetic resonance imaging (MRI), and digital subtraction angiography (DSA). The treatment of HH includes surgery, interventional therapy, and liver transplantation. Studies have shown that propranolol has effect on hemangioma in infants [9], but there is no clear research evidence to support its effectiveness in adults. In general, although the surgical indications for HH have been controversial, it is theoretically possible for all HH patients to undergo surgery if necessary. Especially with the development of laparoscopic techniques and instruments, surgery remains the first-line treatment for HH patients.

However, while the surgery brings a higher cure rate, it also produces some serious complications, such as severe anemia or liver failure [10]. It is recognized that the modified Clavien-Dindo classification [11] is an excellent criterion for evaluating short-term complications after surgery. Therefore, based on the modified Clavien-Dindo classification, this study aims to analyze the risk factors for short-term complications after HH surgery. Our conclusion could provide clinicians with a reference for making individualized treatment decisions, reducing the risk of surgery and the incidence of severe complications.

## Materials and methods

### Study population

We retrieved the information of patients admitted with HH as the first diagnosis from January 2011 to December 2020 in the medical record system of the First Affiliated Hospital of Air Force Medical University. A total of 1155 cases (ICD Code D18, D18.016, D18.019, D18.053, K76.452). Inclusion criteria are as follows: (1) Only underwent surgery; (2) Liver function Child A/B; (3) No history of malignancy; (4) Postoperative pathology of HH (histological type 9120/0, 9121/0, 9131/0). The exclusion criteria are as follows: (1) Only underwent non-surgical treatment; (2) Underwent interventional therapy before surgery; (3) Combined with malignant tumors; (4) Incomplete patient information. Finally, a total of 596 patients were enrolled, including 165 males (27.7%) and 431 females (72.3%).

This study has been approved by the Medical Ethics Committee of the First Affiliated Hospital of the Air Force Medical University (KY20222320-C-1). Data collection and analysis conformed to the standards of the Declaration of Helsinki.

### Definitions and variables

According to the modified Clavien-Dindo classification, surgical complications within 30 days were categorized into seven grades. Also, Grade II/III/IV/V required significant attention from clinicians, such as blood transfusion, closed thoracic drainage, and even secondary surgery, while patients with grade I or no complications only require usual care. Based on these, patients were analyzed in two groups: the Major Group included patients with Grade II/III/IV/V, and the Minor Group included patients with Grade I and no complications. We had detailed records of multiple complications in some patients, but the classification was based on the highest grade. Data collection was performed by three trained clinicians, who stratified and grouped patients. Differences were then checked by the Statistics Department.

We collected the data of each as follows: gender, age, body mass index (BMI), symptoms, comorbidities, history of abdominal surgery, hepatitis B, tumor size, tumor number, location, alanine aminotransferase (ALT), aspartate aminotransferase (AST), albumin (ALB), total bilirubin (TB), sCRE, platelet count (PLT), prothrombin time (PT), combined resection, surgical method, operative duration, inflow control, IBL, blood transfusion, and applied to subsequent statistical analysis. Age at surgery was divided into two groups as a categorical variable, namely < 40 years or ≥ 40 years. BMI was also grouped into < 28 kg/m^2^ or ≥ 28 kg/m^2^. Comorbidities included diabetes, liver cyst, fatty liver, anemia, and hypertension. As long as patients were diagnosed with one of them, this variable counted as “yes”. Combined resection refers to the simultaneous removal of the gallbladder or spleen during HH surgery. The surgical method was categorified into open and laparoscopic surgery. Operative duration was divided into two groups, ≥ 200 min and < 200 min. Generally, IBL exceeding 800 ml in a short time will cause hemodynamic instability or even hemorrhagic shock. Therefore, 800 ml was used as the cut-off value to divide IBL into two groups, ≥ 800 ml and < 800 ml. In addition, based on receiver operating characteristic analysis, we calculated the optimal cutoff value of tumor size to be 9 cm, and grouped it accordingly, ≥ 9 cm and < 9 cm. Location, as a categorical variable, was divided into four groups, left lobe, right lobe, bilateral, and caudate lobe.

### Statistical analysis

Continuous variables with normal distribution were described as mean ± standard deviation, and t-test was used to test for differences between groups. Continuous variables with skewed distribution were described as median (Q1-Q3), and differences between groups were tested using the Mann–Whitney U test. Categorical variables were presented as an absolute number (%), and comparisons were made using the χ^2^ test or Fisher’s exact test. Then, logistic regression analysis was performed for variables with *P* < 0.1 to identify independent risk factors of surgical complications (Grade II/III/IV/V) [12]. At the same time, IBL is recognized as a variable affecting the prognosis of surgical patients, so we also performed univariate and multivariate analyses on IBL. Results were considered significant at *P* < 0.05. All statistical tests were two-sided and were carried out using the SPSS 26.0 software (IBM, Armonk, NY, USA).

## Results

### Clinical characteristics

**Fig. 1:**
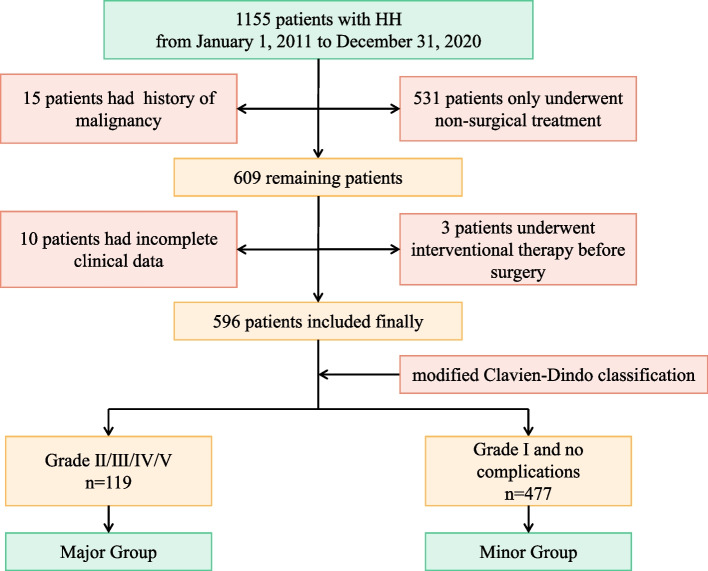
The flowchart of patient inclusion/exclusion criteria and grouping

The flowchart of patient inclusion/exclusion criteria and grouping is shown in Fig. 1. We enrolled 596 HH patients for this study: 119 were assigned to the Major Group (Grade II/III/IV/V, Table 1) and 477 were assigned to the Minor Group (Grade I and no complications), with a median age of 46.0 years (range, 22–75 years). And clinical characteristics of the patients in these two groups are shown in Table 2. No significant differences in gender, BMI, symptoms, comorbidities, history of abdominal surgery, hepatitis B, tumor number, or location between the two groups were found (*P* > 0.05). Moreover, there were no significant differences in ALT, AST, ALB, TB, PLT, or PT between the two groups (*P* > 0.05). However, there were marked differences in age, tumor size, and sCRE (*P* < 0.05).

**Table 1:** Surgical complications based on the modified Clavien-Dindo classification

	Complication	Total
Grade II	Anemia; 67 Hypoalbuminemia; 49 Ascites; 10 Bile leakage; 5 Wound hemorrhage; 2	105
Grade IIIa	Pleural effusion; 7 Wound infection; 1	11
Grade IIIb	Wound hemorrhage; 5	
Grade IVa	Wound hemorrhage; 2	3
Grade IVb	ARDS; 1	
Grade V	Death; 0	0

**Table 2:** Clinical characteristics of included patients

Variables	Major Group(*n* = 119)	Minor Group(*n* = 477)	*P* value
Gender			0.500
male	30(25.2%)	135(28.3%)	
female	89(74.8%)	342(71.7%)	
Age			**0.012**
≥ 40 years	100(84.0%)	348(73.0%)	
< 40 years	19(16.0%)	129(27.0%)	
BMI			0.402
≥ 28 kg/m^2^	7(5.9%)	39(8.2%)	
< 28 kg/m^2^	112(94.1%)	438(91.8%)	
Symptoms			0.884
Yes	53(44.5%)	216(45.3%)	
No	66(55.5%)	261(54.7%)	
Comorbidities			0.229
Yes	25(21%)	78(16.4%)	
No	94(79%)	399(83.6%)	
History of abdominal surgery			0.833
Yes	26(21.8%)	100(21.0%)	
No	93(78.2%)	377(79.0%)	
Hepatitis B			0.877
Yes	8(6.7%)	34(7.1%)	
No	111(93.3%)	443(92.9%)	
Tumor size			**< 0.001**
≥ 9 cm	83(69.7%)	171(35.8%)	
< 9 cm	36(30.3%)	306(64.2%)	
Tumor number			0.553
Solitary	58(48.7%)	247(51.8%)	
Multiple	61(51.3%)	230(48.2%)	
Location			0.219
Left lobe	31(26.1%)	170(35.6%)	
Right lobe	58(48.7%)	192(40.3%)	
Bilateral	27(22.7%)	102(21.4%)	
Caudate lobe	3(2.5%)	13(2.7%)	
ALT, IU/L	15(11–23)	17(12–25)	0.978
AST, IU/L	18(15–23)	18(15–23)	0.155
ALB, g/L	44.1 ± 3.6	44.5 ± 3.7	0.350
TB, μmol/L	13.2(11–16.7)	12.6(10.2–16.3)	0.118
sCRE, μmol/L	80(70–87)	82(74.6–91)	**0.032**
PLT, × 10^9^/L	187(150–235)	183(146–226)	0.688
PT, s	11(10.6–11.6)	11.1(10.6–11.6)	0.715

### Operation data

The operation data of the patients in these two groups are shown in Table 3. No significant differences in combined resection, surgical method, and inflow control (*P* > 0.05). However, there were marked differences in operative duration, IBL, and blood transfusion (*P* < 0.05).

**Table 3:** Operation data of included patients

Variables	Major Group(*n* = 119)	Minor Group(*n* = 477)	*P* value
Combined resection			0.106
Yes	48(40.3%)	155(32.5%)	
No	71(59.7%)	322(67.5%)	
Surgical method			0.070
Laparoscopic	23(19.3%)	131(27.5%)	
Open	96(80.7%)	346(72.5%)	
Operative duration			**< 0.001**
≥ 200 min	65(54.6%)	85(17.8%)	
< 200 min	54(45.4%)	392(82.2%)	
Inflow control^a^			0.151
Yes	76(63.9%)	337(70.6%)	
No	43(36.1%)	140(29.4%)	
IBL			**< 0.001**
≥ 800 ml	69(58.0%)	88(18.4%)	
< 800 ml	50(42.0%)	389(81.6%)	
Blood transfusion			**< 0.001**
Yes	60(50.4%)	87(18.2%)	
No	59(49.6%)	390(81.8%)	

^a^ Pringle’s inflow control was used to reduce blood loss by alternating 15 min of ischemia with 5 min of reperfusion

### Risk factors for Grade II/III/IV/V

Multivariate analysis was performed using binary logistic regression model including all variables with *P* < 0.1 in the previous analysis. The Box-Tidwell method was used to test for a linear relationship between the logit-transformed values of continuous independent and dependent variables. Ten samples with studentized residuals greater than 2.5 times the observed standard deviation were retained in the analysis. Finally, the obtained Logistic model was statistically significant, χ^2^ = 114.511, *P* < 0.001. The model was able to correctly classify 81.9% of the study subjects. Among the 7 independent variables included in the model, operative duration, IBL, tumor size, and sCRE were statistically significant (*P* < 0.05).

The results showed that with operative duration (OR = 2.900; 95% CI: 1.746–4.815; *P* < 0.001), IBL (OR = 2.389; 95% CI: 1.289–4.427; *P* = 0.006), and tumor size (OR = 2.466; 95% CI: 1.520–4.001; *P* < 0.001) increased the risk of Grade II/III/IV/V surgical complications. Conversely, sCRE (OR = 0.984; 95% CI: 0.968–0.999; *P* = 0.039) decreased the risk (Table 4).

**Table 4:** Risk factors for Grade II and above (Method = Enter)

Variables	B	Wald	*P* value	OR	95%CI
Age	0.429	2.118	0.146	1.536	0.862–2.738
Surgical method	0.186	0.398	0.528	1.204	0.677–2.143
Operative duration	1.065	16.928	**< 0.001**	2.900	1.746–4.815
IBL	0.871	7.658	**0.006**	2.389	1.289–4.427
Blood transfusion	0.305	0.968	0.325	1.356	0.739–2.490
Tumor size	0.903	13.361	**< 0.001**	2.466	1.520–4.001
sCRE	-0.017	4.274	**0.039**	0.984	0.968–0.999

### Risk factors for IBL

Univariate analysis showed that tumor size, location, PT, combined resection, surgical method, and operative duration were significant variables (*P* < 0.05). Next, variables with *P* < 0.1 were included in multivariate analysis. Results showed that location, PT, and combined resection were no longer statistically significant. And tumor size (OR = 3.963; 95% CI: 2.511–6.256; *P* < 0.001), surgical method (OR = 1.960; 95% CI: 1.074–3.576; *P* = 0.028), and operative duration (OR = 7.967; 95% CI: 4.885–12.994; *P* < 0.001) increased the risk of IBL (Table 5).

**Table 5:** Risk factors for IBL

				Multivariate (Method = Entered)		
Variables	< 800 ml(*n* = 439)	≥ 800 ml(*n* = 157)	Univariate (*P* value)	OR	95%CI	*P* value
Gender (male/female)	122/317	43/114	0.923			
Age (≥ 40/ < 40)	322/117	126/31	0.087	1.151	0.686–1.930	0.594
BMI (≥ 28/ < 28)	37/402	9/148	0.280			
Symptoms (Y/N)	200/239	69/88	0.728			
Comorbidities (Y/N)	74/365	29/128	0.646			
History of abdominal surgery (Y/N)	91/348	35/122	0.680			
Hepatitis B (Y/N)	32/407	10/147	0.699			
Tumor size (≥ 9/ < 9)	142/297	112/45	**< 0.001**	3.963	2.511–6.256	**< 0.001**
Tumor number (Solitary/Multiple)	225/214	80/77	0.949			
Location (Left/Right/Bilateral/Caudate)	173/168/86/12	28/82/43/4	**< 0.001**	1.675	0.951–2.950	0.074^*^
ALT	17(13–26)	15(12–21)	0.371			
AST	18(16–23)	17(14–22)	0.742			
ALB	44.4 ± 3.5	44.4 ± 4.0	0.994			
TB	12.5(10.1–16.3)	13.7(10.5–17.2)	0.243			
sCRE	81(74–90)	82(75–91)	0.880			
PLT	184(149–229)	184(141–221)	0.223			
PT	11.0(10.5–11.5)	11.2(10.7–11.8)	**0.009**	1.299	0.984–1.715	0.065
Combined resection (Y/N)	130/309	73/84	**< 0.001**	0.987	0.619–1.574	0.955
Surgical method (Laparoscopic/Open)	129/310	25/132	**0.001**	1.960	1.074–3.576	**0.028**
Operative duration (≥ 200/ < 200)	59/380	91/66	**< 0.001**	7.967	4.885–12.994	**< 0.001**
Inflow control (Y/N)	302/137	111/46	0.657			

^*^ In the multivariate analysis, left lobe was used as the reference, and here was the result of right lobe

## Discussion

In this study, 54% of the 1155 patients admitted to the hospital were treated with surgery. HH surgical indications are controversial, but surgery remains a high priority in some conditions. First, the presence of symptoms is an important indication. In this study, the proportion of patients with symptoms in the two groups reached 44.5% and 45.3% respectively, which was similar to the proportion in previous studies [13] (TAE 26/53, MWA 44/82). Second, continued growth in tumor size, or even rapid growth in the short term, may induce symptoms or lead to complications. Of the surgical patients we recorded, 32% of patients underwent surgery because of tumor enlargement. In addition, suspected hemangioma with uncertain clinical diagnosis is also considered as an indication for surgery, especially in patients with hepatitis, cirrhosis, or other malignancies. Finally, psychiatric symptoms are also one of the important surgical indications. Some patients have mental problems such as restlessness, anxiety, or depression due to pessimism about disease progression and prognosis. Therefore, this priority drives us to value the impact of its complications on the patient while taking advantage of the high cure rate of surgery. In our and previous studies [14], the proportion of Grade II/III/IV/V complications was close to 20%, which also supports this view.

To reduce the impact of surgical complications on patients, we investigated risk factors for Grade II/III/IV/V complications after HH surgery. Considering the results of two Logistic regressions, we concluded that tumor size, IBL, and operative duration were independent risk factors for grade II and above complications, and sCRE was an independent protective factor. Tumor size, surgical method, and operative duration were independent risk factors for IBL. In this study, the proportion of ≥ 9 cm HH in the Major group was significantly higher than that in the Minor group (69.7 vs. 35.8%, *P* < 0.001). In the operation data, the proportion of HH patients with IBL ≥ 800 ml in the Major group was also significantly higher than that in the Minor group (58.0 vs. 18.4%, *P* < 0.001). We think the reasons may be as follows. First, larger tumors result in larger surgical wounds, which are more difficult for patients to heal and lead to more IBL. Second, IBL can lead to hemodynamic instability, with liver function ranging from mild abnormalities to liver failure. IBL can also induce systemic inflammatory response and hemodynamic instability, which increase the risk of various postoperative complications [15]. There are studies with similar conclusions to ours, but not identical. Mohamed Abdel Wahab et al. [16] showed that HH size does significantly affect IBL, median IBL in > 10 cm HH patients was significantly higher than in < 10 cm HH patients (575 vs. 300 ml, *P* = 0.007). But the difference is that they do not think it will affect the complications rate (23.6 vs. 20.8%, *P* = 0.690). Jian Dong et al. [14] showed that tumor size was not an independent risk factor for complications (8.25 vs. 8.38 cm, *P* = 0.748). The variable IBL was also not statistically significant in the multivariate analysis of their study (RR = 2.217; 95% CI: 0.765–5.127; *P* = 0.112). However, in univariate analysis, IBL was significantly higher in the complication group (956.7 vs. 424.2 ml, *P* = 0.013). The part of results of these two literatures on IBL is the same as ours, but the effect of IBL on complications is different. We speculate that this difference is due to the different selection of outcome events. They focused on risk factors for all complications (Grade I/II/III/IV/V), and we focused on Grade II/III/IV/V. Because the treatment of Grade I complications is only routine intervention, and the treatment of Grade II complications becomes complicated, including blood transfusion and total parenteral nutrition [11]. We think it is more clinically meaningful to exclude Grade I. And from the latter results, we did find that the incidence of Grade II/III/IV/V complications in group B (10-15 cm) was higher than that in group A (5-10 cm, 16/19 vs. 21/43). Grade III/IV/V complications have similar outcomes between the two groups (4/19 vs. 1/43, *P* = 0.013). In fact, these were the same as our conclusions. Therefore, controlling IBL during HH surgery is the primary concern of hepatobiliary surgeons, and the amount of bleeding is directly related to the risk of postoperative Grade II/III/IV/V complications. Our findings clearly demonstrate this relationship.

Operative duration ≥ 200 min HH patients in the Major group was significantly longer than in the minor group (54.6 vs. 17.8%, *P* < 0.001). Operative duration is often related to many factors, such as the degree of intra-abdominal adhesions, the proficiency of the surgeon, and the individual anatomy of the patient. We think that it does not constitute a causal relationship with postoperative complication rates, and the longer operative duration is likely to be caused by other risk factors. For example, larger tumor increases the difficulty of surgery, thus prolonging the operative duration. Moreover, without considering the influence of other risk factors, prolonged operative duration itself will increase the air exposure time of the patient's internal organs and increase the probability of infection. The proportion of laparoscopic surgery in the ≥ 800 ml group was significantly lower than that in the < 800 ml group (25/132 vs. 129/310, *P* = 0.001). As shown in our findings, laparoscopic surgery is known to reduce IBL and postoperative complication rates in patients at the expense of increased procedural difficulty. Chen Yan et al. [17] also reported in the study that laparoscopic surgery is superior to open surgery in controlling IBL. The authors believe that laparoscopic surgery can provide a clearer global view and a magnified local view, which is beneficial for identifying blood vessels. And there was no statistically significant difference in the probability of postoperative Grade II/III/IV/V complications between the two surgical methods. In addition, a meta-analysis [18] also showed that, in older adults, laparoscopic surgery was superior to open surgery in terms of bleeding control and postoperative complication rates. On the one hand, the surgical wound of open surgery is larger. On the other hand, the application of pneumoperitoneum makes the venous pressure lower. A study [19] has shown that patients undergoing laparoscopic surgery have a significantly shorter hospital stay, which is one of its advantages. However, as HH becomes larger and more difficult to operate, the advantages of laparoscopic surgery are no longer obvious. In the face of giant HH, clinicians are more inclined to choose open surgery to avoid accidents.

Of all the independent risk factors, we were most concerned with IBL and sCRE. In our results, preoperative sCRE level was an independent protective factor for postoperative complications (OR = 0.984; 95% CI: 0.968–0.999; *P* = 0.039). sCRE is naturally produced by the human body and is closely related to total muscle mass. The increase is generally seen in renal dysfunction, and the decrease is seen in liver dysfunction. The statistical results seem to suggest that the Major group with relatively lower sCRE had slightly worse liver function than the Minor group. This difference in liver function results in inconsistent complication rates. However, sCRE is generally used as an important evaluation index of renal function, and its relationship with complications of liver surgery is rarely reported. The underlying mechanism for lower sCRE levels leading to higher complication rates remains unclear. And it is not known whether our inferences relying on statistical results are clinically meaningful. A study [20] about sCRE levels on short- and long-term complications of cardiac surgery showed that it was an independent predictor of mortality (short-: HR = 1.59, 95% CI: 1.38–1.83, *P* = 0.0027; long-: HR = 1.46, 95% CI: 1.32–1.62, *P* = 0.0001). But their cut-off value of sCRE, 115 μmol/L, was set higher, which was already past the line between normal and outliers. Another study [21] about factors related to intraoperative bleeding in liver transplantation showed that sCRE had a weaker correlation (correlation coefficient = 0.152, *P* < 0.001). In addition, some scholars [22] have directly proposed that the risk factors for bleeding during hepatectomy for primary liver malignancies include sCRE. Although the numerical difference in sCRE between the two groups was not large in our study (80 vs. 82, *P* = 0.032), the statistical difference suggested that the mechanism behind this is worth exploring and may have potentially huge clinical value.

Last but not least, the mechanism of propranolol has not been fully elucidated and may be related to the promotion of vasoconstriction, inhibition of angiogenesis, and inactivation of the renin-angiotensin system [23]. At least so far, there are few studies of oral propranolol in the treatment of HH in adults. From the perspective of the treatment of infants, it is safe and effective, with few adverse reactions, and is expected to become a first-line drug for the treatment of severe hemangiomas. This suggests that there is still a huge development space for β-blockers represented by propranolol, and further exploration of their mechanism in the future may fill the gap in adult HH drug treatment.

There are some limitations to our study. First of all, this is a retrospective study. The data source is mainly the medical record system of our hospital, and there may be some missing content in the records. Second, the long time span of our collection makes it impossible for our study to rule out the influence of surgical technique development itself. The influence also even includes updates to blood test technology of our hospital. Third, even though the source of patients in our center covers multiple provinces in China, this study is essentially a single-center study.

This study also has some advantages. A total of 596 patients were finally included. Clear and strict inclusion and exclusion criteria and a large sample size enhance the credibility of our conclusions. About the selection of outcome events, we referred to the modified Clavien-Dindo classification, which is recognized as one of the most authoritative and credible standards in the world for evaluating postoperative complications including gastrointestinal and liver surgery.

In conclusion, our study shows that operative duration, IBL, tumor size, and surgical method are independent risk factors that should be paid attention to in HH surgery. Besides them, sCRE is controversial as an independent protective factor for postoperative complications. We expect more scholars to concern about this phenomenon, because it may not only benefit liver surgery but also explore the mechanism of interaction between liver and kidney. Under the premise of the limited application of interventional therapy and lack of drug therapy, we should further optimize the details of surgery to speed up patient recovery and reduce pain.

## Data Availability

The datasets used and analyzed during the current study are available from the corresponding author upon reasonable request.

## Abbreviations

HH: Hepatic hemangioma
IBL: Intraoperative blood loss
sCRE: Serum creatinine
CT: Computed tomography
MRI: Magnetic resonance imaging
DSA: Digital subtraction angiography
ICD: International classification of diseases
BMI: Body mass index
ALT: Alanine aminotransferase
AST: Aspartate aminotransferase
ALB: Albumin
TB: Total bilirubin
PLT: Platelet count
PT: Prothrombin time
OR: Odds ratio
CI: Confidence interval
TAE: Transcatheter arterial embolization
MWA: Microwave ablation

## References

[1] Hasan HY, Hinshaw JL, Borman EJ, Gegios A, Leverson G, Winslow ER (2014). Assessing normal growth of hepatic hemangiomas during long-term follow-up. Jama Surg.

[2] Karhunen PJ (1986). Benign hepatic tumours and tumour like conditions in men. J Clin Pathol.

[3] Akbulut S, Yilmaz M, Kahraman A, Yilmaz S (2013). Bilateral lower limb edema caused by compression of the retrohepatic inferior vena cava by a giant hepatic hemangioma. Int Surg.

[4] Aydin C, Akbulut S, Kutluturk K, Kahraman A, Kayaalp C, Yilmaz S (2013). Giant hepatic hemangioma presenting as gastric outlet obstruction. Int Surg.

[5] Leon M, Chavez L, Surani S (2020). Hepatic hemangioma: What internists need to know. World J Gastroentero.

[6] Glinkova V (2004). Hepatic haemangiomas: possible association with female sex hormones. Gut.

[7] Hoekstra LT, Bieze M, Erdogan D, Roelofs JJ, Beuers UH, Gulik TMV (2014). Management of giant liver hemangiomas: an update. Expert Rev Gastroent.

[8] Bioulac-Sage P, Laumonier H, Laurent C, Blanc J, Balabaud C (2008). Benign and malignant vascular tumors of the liver in adults. Semin Liver Dis.

[9] Léauté-Labrèze C, Harper JI, Hoeger PH (2017). Infantile haemangioma. The Lancet.

[10] Søreide JA, Deshpande R (2021). Post hepatectomy liver failure (PHLF) – Recent advances in prevention and clinical management. Eur J Surg Oncol.

[11] Clavien PA, Barkun J, de Oliveira ML, Vauthey JN, Dindo D, Schulick RD, de Santibañes E, Pekolj J, Slankamenac K, Bassi C (2009). The Clavien-Dindo classification of surgical complications. Ann Surg.

[12] Akbulut S, Sahin TT, Yilmaz S (2020). Comment on pediatric living donor liver transplantation decade progress in Shanghai: characteristics and risks factors of mortality. World J Gastroentero.

[13] Shi Y, Song J, Ding M, Tang X, Wang Z, Chi J, Wang T, Ji J, Zhai B (2020). Microwave ablation versus transcatheter arterial embolization for large hepatic hemangiomas: clinical outcomes. Int J Hyperther.

[14] Dong J, Zhang M, Chen J, Ma F, Wang H, Lv Y (2015). Tumor size is not a criterion for resection during the management of giant hemangioma of the liver. Eur J Gastroen Hepat.

[15] Rahbari NN, Garden OJ, Padbury R, Brooke-Smith M, Crawford M, Adam R, Koch M, Makuuchi M, Dematteo RP, Christophi C (2011). Posthepatectomy liver failure: A definition and grading by the International Study Group of Liver Surgery (ISGLS). Surgery.

[16] Abdel Wahab M, El Nakeeb A, Ali MA, Mahdy Y, Shehta A, Abdulrazek M, El Desoky M, Abdel Wahab R (2018). Surgical management of giant hepatic hemangioma: single center’s experience with 144 patients. J Gastrointest Surg.

[17] Yan C, Li B, Sun X, Yu D (2021). Laparoscopic hepatectomy is superior to open procedures for hepatic hemangioma. Hepatob Pancreat Dis.

[18] Hildebrand N, Verkoulen K, Dewulf M, Heise D, Ulmer F, Coolsen M (2021). Short-term outcomes of laparoscopic versus open hepatectomy in the elderly patient: systematic review and meta-analysis. HPB.

[19] Xie Q, Chen Z, Zhao Y, Gu H, Geng X, Liu F. Outcomes of surgery for giant hepatic hemangioma. BMC Surg. 2021;21(1):186. 10.1186/s12893-021-01185-4 PMC8033692 33832476

[20] Bernardi MH, Schmidlin D, Schiferer A, Ristl R, Neugebauer T, Hiesmayr M, Druml W, Lassnigg A (2015). Impact of preoperative serum creatinine on short- and long-term mortality after cardiac surgery: a cohort study. Brit J Anaesth.

[21] Eghbal MH, Samadi K, Khosravi MB, Sahmeddini MA, Ghaffaripoor S, Ghorbani M, Shokrizadeh S (2019). The Impact of Preoperative Variables on Intraoperative Blood Loss and Transfusion Requirements During Orthotopic Liver Transplant. Exp Clin Transplant.

[22] Imai D, Maeda T, Wang H, Shimagaki T, Sanefuji K, Kayashima H, Tsutsui S, Matsuda H, Yoshizumi T, Mori M (2021). Risk factors for and outcomes of intraoperative blood loss in liver resection for hepatocellular tumors. Am Surgeon.

[23] Ji Y, Chen S, Xu C, Li L, Xiang B (2015). The use of propranolol in the treatment of infantile haemangiomas: an update on potential mechanisms of action. Brit J Dermatol.

